# Vaccination an Unhomœopathic Measure*Read before the Brooklyn Hahnemannian Union, December, 1897.

**Published:** 1898-02

**Authors:** B. Fincke

**Affiliations:** Brooklyn, N. Y.


### VACCINATION AN UNHOMŒOPATHIC MEASURE.*

B. Fincke, M. D., Brooklyn, N. Y.

It is true, as said in the article on "Vaccination" in the September number of The Homœopathic Physician, page 367, that the fourth American edition of Stratten's translation of the fourth German edition of *The Organon* of 1829, provided by the American editors with improvements and additions from the fifth German edition of 1833, on page 75 contains the quotation about cow-pox in relation to small-pox as being homœopathic to it in appearance and action. In Dudgeon's translation, however, it is not to be found, for the reason that it is also not contained in the fifth German edition. On examination it is found that the pages 59–89 in the American Stratten, giving numerous examples of *homœopathia involuntaria* in allopathic practice which were taken from the fourth German edition, have been left out in the fifth by Hahnemann himself. Thus, also, cases of scabies curing diseases in the third edition have been left out by him afterward because he deemed them inappropriate. On the other hand, the sections relating to the resemblance of cow-pox to small-pox have been inserted in the text of *The Organon* ever since 1810 up to 1833 as examples that similar diseases overcome one another on account of their homœopathicity.

To this first follows a second quotation from a letter of Hahnemann, expressing his horror at the attacks on cow-pox vaccination from the year 1825. To this can a third quotation be added from a letter to Stapf December 22d, 1825, in which Hahnemann is horrified at the opposition to Jenner's vaccination in England "after Jenner's protective cow-pox has proved successful everywhere." And finally, in 1829 he reverts to small-pox and cow-pox in their instantaneous infection when "at its inoculation the morbid fluid comes in contact with the exposed nerve in the bloody scratch." (See *Chron. Dis.*, page 43, German

* Read before the Brooklyn Hahnemannian Union, December, 1897.

edition.) This is all that could be gleaned thus far from the writings of Hahnemann, numerous as they are.

What follows from these quotations? Does it follow that the inoculation of the cow-pox, genuine or spurious, as used nowadays, is a homœopathic measure? Is it on that account obligatory upon every homœopathic physician to practice vaccination as prescribed by law? Is the mode of inoculation of matter which is neither original cow-pox nor inoculated human cow-pox, but animal cow-pox of questionable origin, hence altogether different from Jenner's vaccination, to be considered authoritative vaccination? Is it a heresy to say that this kind of vaccination is only a conventional substitute for inoculation with vaccine, which alone can be called vaccination, lacking all claim to resemblance to small-pox and cow-pox, and therefore unhomœopathic in appearance and action? Have the homœopathic physicians fallen so low that still, after all the experience of many years, they hang on to that contemptible tail of the allopathic profession, inoculation of animal infectious matter? And can there be any doubt about it, that this kind of vaccination is incompatible with and antagonistic to homœopathic practice?

Even falling back upon the authority of our beloved Hahnemann cannot strengthen the belief in vaccination, for he nowhere has taught and inculcated vaccination in any form, and is perfectly silent about it, mentioning only as a precaution the giving of a dose of Sulphur after the operation to prevent any deleterious action. This silence is still more ominous for the friends of vaccination, since there is no mention of small-pox as a consequence of one of the original miasms. We do not know whether he had vaccinated himself, though we may, from the forcible expressions in the letters quoted, assume that he did. Nor do we know why he passed over such an important subject without alluding to the prophylactic measure of preventing small-pox when he mentioned Belladonna as a preventive of scarlatina, and used the resemblance of small-pox to cow-pox as a confirmation of the homœopathic law. How, then, can any homœopathician favoring vaccination base his adherence to it upon Hannemannian authority, and blindly follow the allopathic crowd in its senseless clamor? Of course every physician has a right to his opinions, and no fault can be found with him if they are founded upon reason. But to base this vile measure upon the teachings of homœopathics by Hahnemann must be denounced as an irresponsible departure from it, and such practice declared to be without right and reason in a homœopathician. This barbaric inoculation of a poison for a protection from small-pox has originally come from the barbarism of the Orient, and the Occident has taken it up as the salvation from the epidemics of small-pox which from time to time scourge the nations. There is ample proof that it does not furnish the protection which is claimed for it; quite the contrary is the experience, according to the testimony of creditable persons and authorities, that the inoculation renders the vaccinated and re-vaccinated more susceptible to the infection with small-pox, so that they are the first to be affected in an epidemic.

Strange that Hahnemann, the arch-enemy of allopathy, should be quoted by homœopathicians as a staunch upholder of an entirely unhomœopathic measure in contradiction with his scientific and humane teaching throughout his works. Nobody has a right to load him down with such a monstrous charge, least of all those who bear his name. Admitted that Hahnemann advocated vaccination with cow-pox and practiced it himself, this does not prove anything for doing as he did. Yet his silence after 1829 shows unmistakably that he had changed his mind, or else he would have made a mark of it in his last edition of *The Organon*, in 1833, or afterward, for he lived ten years longer. Once he owned up that his examples of itch curing disease were a mistake, and he left them out in the following editions. If he was so honest in this, why should we not assume that he was equally honest in not recommending vaccination, and give him the benefit of the doubt? There is indeed no "sad blot" on that great work, *The Organon*. Hahnemann, at the time when he was occupied with the bringing out of his immortal work, the *Chronic Diseases*, had more to do than to bother himself with that wretched vaccination question.

Stranger still, that men who know the superior power of the infinitesimals and practice accordingly should stoop to advocate the forcible vaccination of our age. Do they administer their infinitesimal remedies by hypodermal injection? Do they not see that this vile operation introduced by Jenner a hundred years ago has been made the type of allopathic medication, so that at the close of our glorious century they inoculate their pernicious remedies of all sorts directly into the organism? How can they reconcile this unphysiological procedure with their reason, not to speak of their conscience? Nothing more glaringly shows the difference between allopathic and homœopathic practice than this baleful dogma of vaccination. Hahnemann has taught everywhere that medicine, if homœopathic to the case, cannot be given in doses too small; nay, he insisted upon it that the homœopathic remedy should be so prepared by his peculiar method of potentiation that neither the senses nor the physical and chemical tests of science could detect the least presence of drug or matter in it, so that any thought of State supervision could not be entertained. And here a Hahnemannian homœopathician states that "by inference, if not by innuendo, the idea is conveyed that a belief in vaccination and Homœopathy are antagonistic." Neither by inference nor by innuendo this idea has been conveyed, but by plain reasoning upon plain facts. (See *American Homœopathist*, 1886, p. 114; *Journal of Homœop.*, 1889, p. 152; HOM. PHYSICIAN, 1886, p. 83; 1888, p. 544; 1890, p. 304; 1892, pp. 465, 468, 476, 478, 482, 485; 1893, pp. 17, 392, 598; 1894, pp. 85, 163, 274, 283; 1885, pp. 228, 267, 431; 1896, pp. 420, 496; 1897, pp. 226, 325, 347, 371, 375; *Proceed. of I. H. A.*, 1892, pp. 100, 110; 1894, p. 210; 1895, pp. 69, 254; 1896, p. 143; 1897, p. 245.)

Does this vaccinator in his practice ever inoculate or inject the thirteenth, the two hundredth, the thousandth, or even the hundred thousandth potency into the system in the manner now generally adopted by allopathy? Very likely not. Why should he then follow the big crowd which finds its salvation in vaccination, and participate in promoting this wholesale poisoning of the nations under the pretext of stamping out the epidemics of small-pox which in spite of their efforts go on all the same?

Have there not been victims enough to increase their number by the perpetuation of this outrage upon mankind?

To return to the quotation first given, which occurs in the introduction to *The Organon* of 1829 for the last time, it says nothing more than that cow-pox can protect from small-pox only because it is homœopathic, and the latter, appearing only once in a lifetime, is the weaker, if the stronger similar cow-pox supervenes upon it. But even that is not distinctly demonstrated, in 1833 (*Organon*, Section 46), where small-pox is declared to be the stronger disease, and is merely ameliorated by inoculated cow-pox in its maturity. But of protection there is not a trace. Besides, small-pox befalls some individuals several times in a lifetime.

To construe from the foregoing that every follower of Hahnemann, rather every disciple—for there are too many mere followers—is bound to vaccinate and re-vaccinate as is the fashion now, this inference and innuendo passes all understanding. May, then, he who vaccinates and thinks the world of it, do as he pleases, for this is a free country, where, alas! anybody can go to perdition who wants to. But let every one else rise up against that intolerable tyranny, placed like a yoke upon the neck of this free nation, to compel every living child to be vaccinated on penalty of exclusion from an education through the public schools if they do not bow down before this modern Gessler's hat.

It has been stated that cow-pox is syphilis, a question which is not yet settled; that it transfers disease in the sound body, of which there are shocking examples, not one but many; that there are other methods of protection from small-pox which most anti-vaccinationists reject because they come from the homœopathic camp; that water-treatment is an effective means of combating small-pox, of which there is ample testimony; that small-pox can be prevented and cured homœopathically, of which there is no doubt; that it gets well under suitable hygienic management, which is quite reasonable; that general improved sanitary conditions will lessen and mitigate the epidemics, as is a practical experience. All these points are legitimate subjects of scientific research, which are open to all thinking men, and should be carried on with diligence and thoroughness upon homœopathic principles. But compulsory vaccination should be rejected with all the scorn and energy of free men.

*Ceterum censeo macrodosiam esse delendam.*

